# The genome sequence of the Saxon Wasp, *Dolichovespula saxonica *(Fabricius, 1793)

**DOI:** 10.12688/wellcomeopenres.20180.1

**Published:** 2023-10-27

**Authors:** Liam M. Crowley, University of Oxford and Wytham Woods Genome Acquisition Lab, Darwin Tree of Life Barcoding collective, Wellcome Sanger Institute Tree of Life programme, Wellcome Sanger Institute Scientific Operations: DNA Pipelines collective, Tree of Life Core Informatics collective, Darwin Tree of Life Consortium

**Affiliations:** 1 Department of Biology, University of Oxford, Oxford, England, UK

**Keywords:** Dolichovespula saxonica, Saxon Wasp, genome sequence, chromosomal, Hymenoptera

## Abstract

We present a genome assembly from an individual male *Dolichovespula saxonica* (the Saxon Wasp; Arthropoda; Insecta; Hymenoptera; Vespidae). The genome sequence is 221.8 megabases in span. Most of the assembly is scaffolded into 26 chromosomal pseudomolecules. The mitochondrial genome has also been assembled and is 18.97 kilobases in length. Gene annotation of this assembly on Ensembl identified 10,856 protein coding genes.

## Species taxonomy

Eukaryota; Metazoa; Eumetazoa; Bilateria; Protostomia; Ecdysozoa; Panarthropoda; Arthropoda; Mandibulata; Pancrustacea; Hexapoda; Insecta; Dicondylia; Pterygota; Neoptera; Endopterygota; Hymenoptera; Apocrita; Aculeata; Vespoidea; Vespidae; Vespinae; *Dolichovespula*; *Dolichovespula saxonica* (Fabricius, 1793) (NCBI:txid85443).

## Background

The Saxon Wasp, *Dolichovespula saxonica,* is medium-sized (11–18 mm) social wasp in the family Vespidae. It occurs across the Palaearctic from northern and central Europe to northern Asia and Japan ( Archer, 1999). It was first recorded from the UK in Surrey in 1987 ( Allen & Archer, 1989) and has since spread throughout Britain, reaching northwards to Scotland. It is now common and widespread across the UK. *D. saxonica* is very similar in appearance to *D. norwegica*, to which it is closely related. It lacks the pair of red markings on the second gastral tergite which *D. norwegica* often has, and the hairs on the side of the thorax are light brown rather than black.

It can be found across a range of open habitats, including heathland, open woodland and urban environments. It is a eusocial species, forming colonies of up to around 1,800 individuals ( Nadolski, 2012). Overwintered queens emerge and initiate nests around May. Paper nests are typically constructed in aerial situations such as trees and shrubs 2 to 7 m above the ground, although a range of other sites are known including buildings, tree holes and beehives ( Archer, 2006). Workers are produced from early June and males and new queens from early July. Mature nests contain an average of approximately 1300 cells across three to five combs ( Archer, 2006). As with other species in the genus, *D. saxonica* exhibits low effective paternity and relatively high incidence of worker reproduction ( Foster *et al.*, 2001).

*D. saxonica* is a generalist predator, taking a wide variety of prey. It feeds on nectar from a wide range of flowers. Queens are known to visit bilberry flowers in the spring and workers and males are recorded visiting wild angelica, wild parsnip and hogweed. It is able to lay pheromone trails used for foraging and orientation ( Steinmetz & Schmolz, 2003).

The complete genome sequence for this species, along with recent publication of Vespinae genomes, including *Dolichovespula* ( Falk *et al.*, 2022), *Vespa* ( Crowley *et al.*, 2022), and *Vespula* ( Crowley *et al.*, 2021), will facilitate studies into many areas such as the evolution of sociality, reproductive systems and Hymenopteran taxonomy.

## Genome sequence report

**Figure 1.**
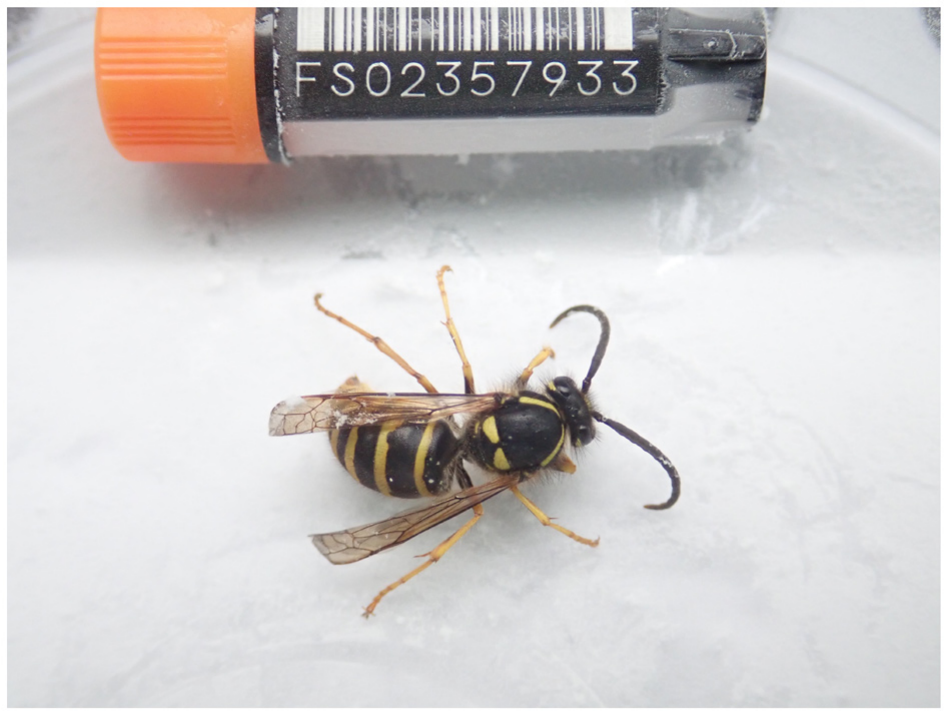
Photograph of the *Dolichovespula saxonica* (iyDolSaxo1) specimen used for genome sequencing.

The genome was sequenced from one male *Dolichovespula saxonica* ( Figure 1) collected from Wytham Woods, Oxfordshire, UK (51.77, –1.33). A total of 27-fold coverage in Pacific Biosciences single-molecule HiFi long reads and 115-fold coverage in 10X Genomics read clouds were generated. Primary assembly contigs were scaffolded with chromosome conformation Hi-C data. Manual assembly curation corrected 288 missing joins or mis-joins, reducing the scaffold number by 55.2%, and increasing the scaffold N50 by 137.4%.

**Figure 2.**
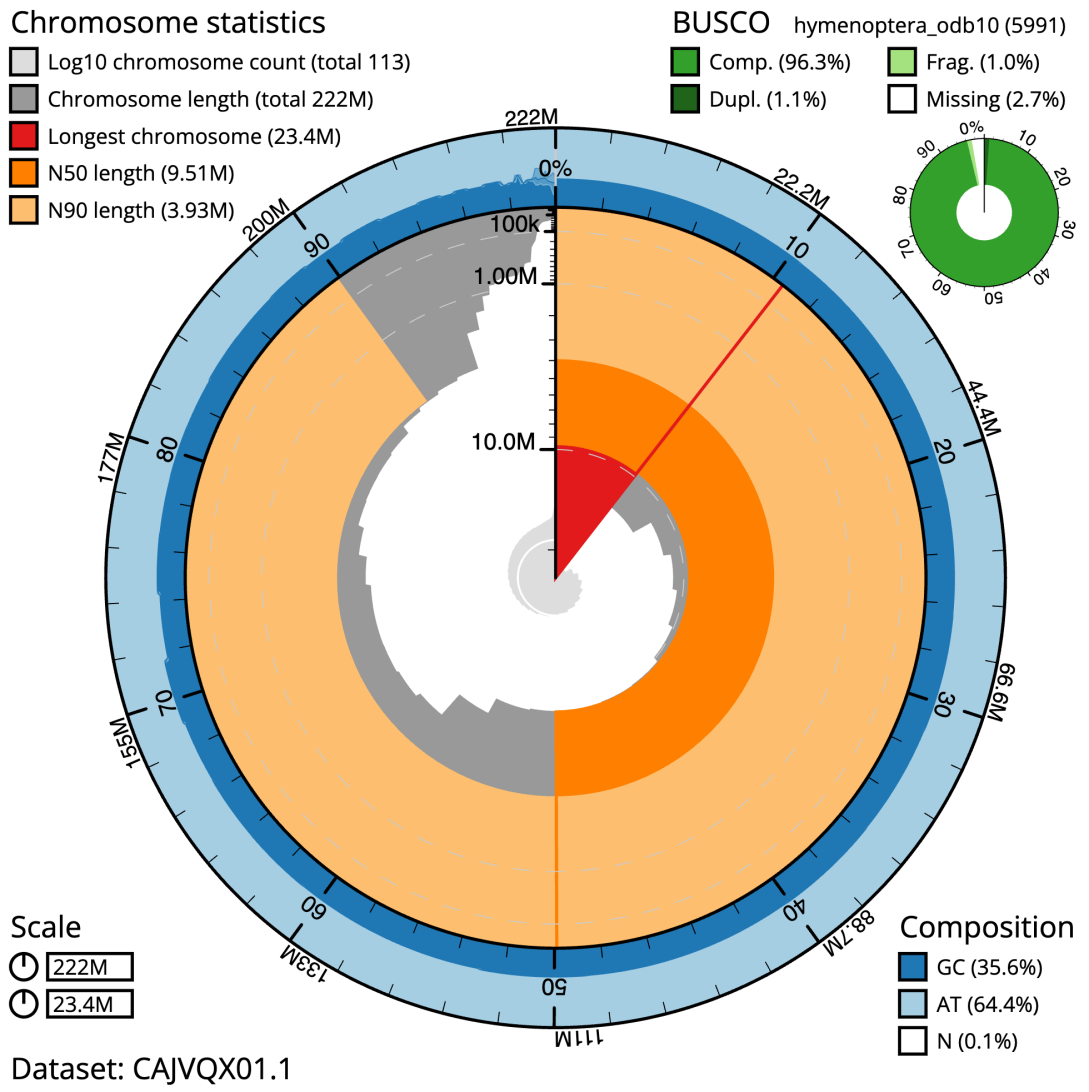
Genome assembly of *Dolichovespula saxonica*, iyDolSaxo1.1: metrics. The BlobToolKit Snailplot shows N50 metrics and BUSCO gene completeness. The main plot is divided into 1,000 size-ordered bins around the circumference with each bin representing 0.1% of the 221,852,803 bp assembly. The distribution of scaffold lengths is shown in dark grey with the plot radius scaled to the longest scaffold present in the assembly (23,378,875 bp, shown in red). Orange and pale-orange arcs show the N50 and N90 scaffold lengths (9,507,211 and 3,928,253 bp), respectively. The pale grey spiral shows the cumulative scaffold count on a log scale with white scale lines showing successive orders of magnitude. The blue and pale-blue area around the outside of the plot shows the distribution of GC, AT and N percentages in the same bins as the inner plot. A summary of complete, fragmented, duplicated and missing BUSCO genes in the hymenoptera_odb10 set is shown in the top right. An interactive version of this figure is available at https://blobtoolkit.genomehubs.org/view/Dolichovespula%20saxonica/dataset/CAJVQX01.1/snail.

**Figure 3.**
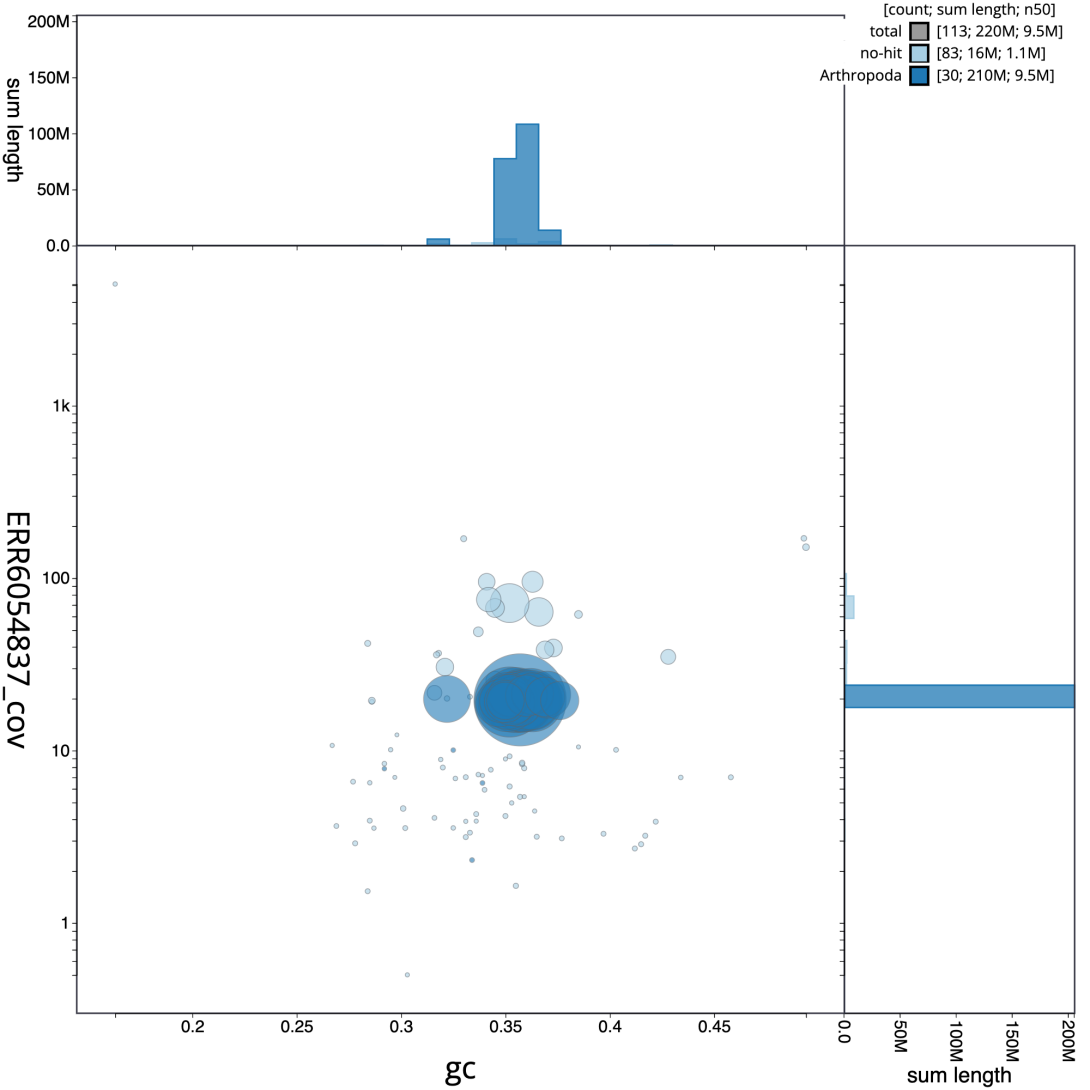
Genome assembly of *Dolichovespula saxonica*, iyDolSaxo1.1: BlobToolKit GC-coverage plot. Scaffolds are coloured by phylum. Circles are sized in proportion to scaffold length. Histograms show the distribution of scaffold length sum along each axis. An interactive version of this figure is available at https://blobtoolkit.genomehubs.org/view/Dolichovespula%20saxonica/dataset/CAJVQX01.1/blob.

**Figure 4.**
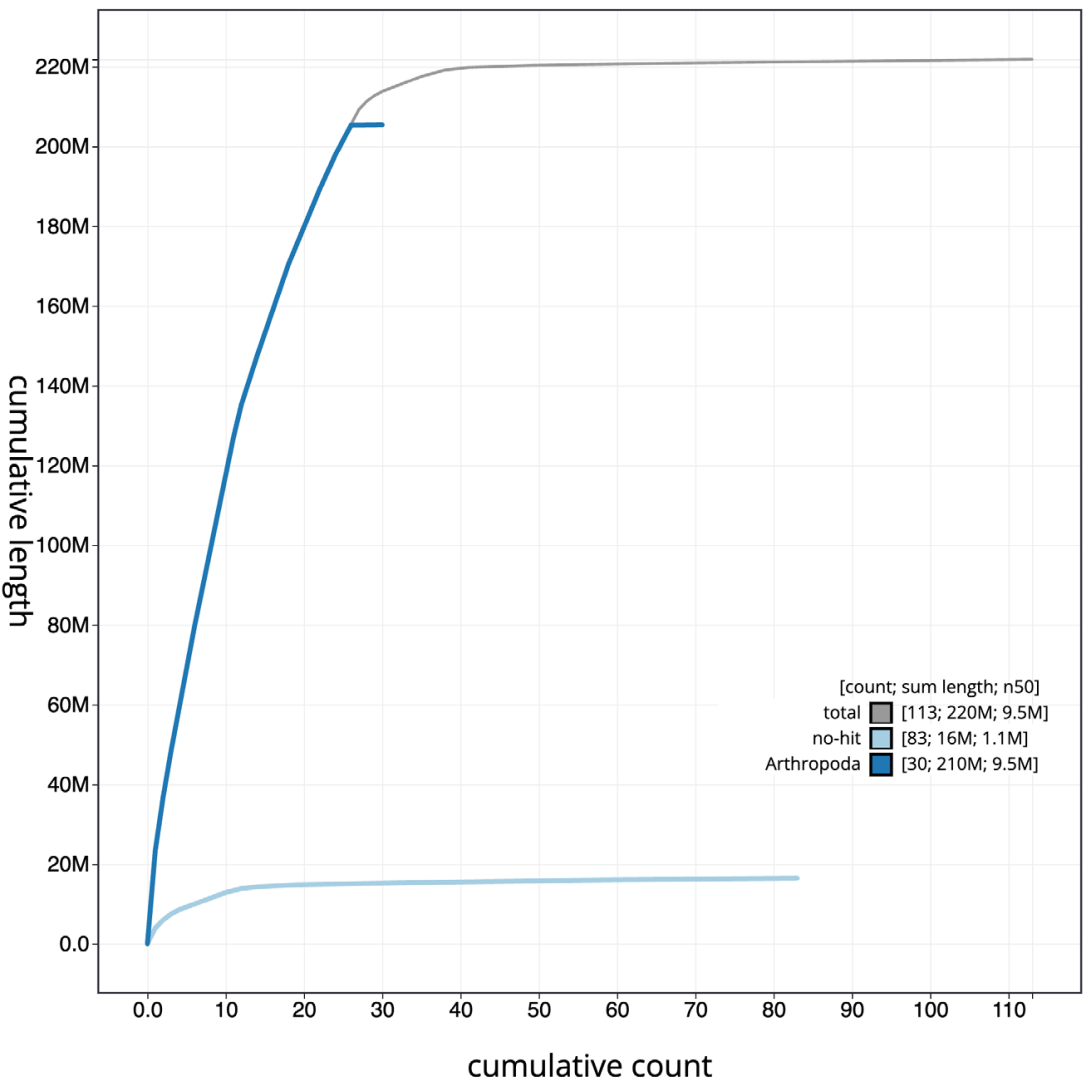
Genome assembly of *Dolichovespula saxonica*, iyDolSaxo1.1: BlobToolKit cumulative sequence plot. The grey line shows cumulative length for all scaffolds. Coloured lines show cumulative lengths of scaffolds assigned to each phylum using the buscogenes taxrule. An interactive version of this figure is available at https://blobtoolkit.genomehubs.org/view/Dolichovespula%20saxonica/dataset/CAJVQX01.1/cumulative.

**Figure 5.**
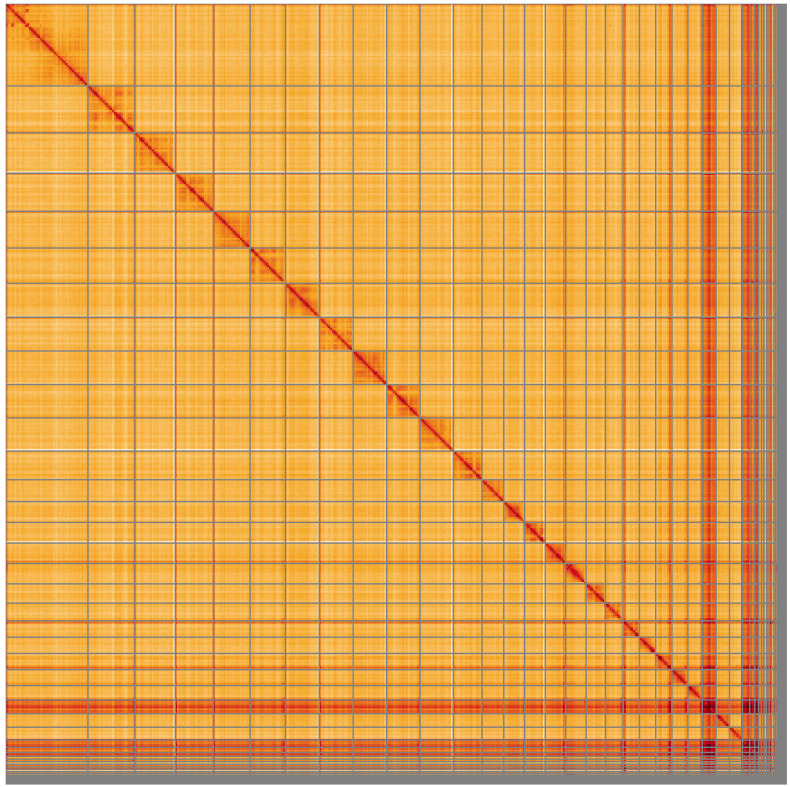
Genome assembly of *Dolichovespula saxonica*, iyDolSaxo1.1: Hi-C contact map of the iyDolSaxo1.1 assembly, visualised using HiGlass. Chromosomes are shown in order of size from left to right and top to bottom. An interactive version of this figure may be viewed at https://genome-note-higlass.tol.sanger.ac.uk/l/?d=EP4az_NHSS-9fl8YSI6n0Q.

The final assembly has a total length of 221.8 Mb in 112 sequence scaffolds with a scaffold N50 of 9.5 Mb ( Table 1). Most (92.54%) of the assembly sequence was assigned to 26 chromosomal-level scaffolds. A summary of the assembly statistics is shown in Figure 2, while the distribution of assembly scaffolds on GC proportion and coverage is shown in Figure 3. The cumulative assembly plot in Figure 4 shows curves for subsets of scaffolds assigned to different phyla. Chromosome-scale scaffolds confirmed by the Hi-C data are named in order of size ( Figure 5; Table 2). The specimen is a haploid male. The mitochondrial genome was also assembled and can be found as a contig within the multifasta file of the genome submission.

**Table 1.** Genome data for *Dolichovespula saxonica*, iyDolSaxo1.1.

Project accession data		
Assembly identifier	iyDolSaxo1.1	
Assembly release date	20201-07-21	
Species	*Dolichovespula saxonica*	
Specimen	iyDolSaxo1	
NCBI taxonomy ID	85443	
BioProject	PRJEB45173	
BioSample ID	SAMEA7520489	
Isolate information	iyDolSaxo1	
Assembly metrics *		*Benchmark*
Consensus quality (QV)	49.2	*≥ 50*
*k*-mer completeness	99.96%	*≥ 95%*
BUSCO **	C:96.3%[S:95.2%,D:1.1%], F:1.0%,M:2.7%,n:5,991	*C ≥ 95%*
Percentage of assembly mapped to chromosomes	92.54%	*≥ 95%*
Sex chromosomes	-	*localised homologous pairs*
Organelles	Mitochondrial genome assembled	*complete single alleles*
Raw data accessions		
PacificBiosciences SEQUEL II	ERR6560800	
10X Genomics Illumina	ERR6054837, ERR6054840, ERR6054838, ERR6054839	
Hi-C Illumina	ERR6054841	
PolyA RNA-Seq Illumina	ERR9434979	
Genome assembly		
Assembly accession	GCA_911387935.1	
*Accession of alternate haplotype*	-	
Span (Mb)	221.8	
Number of contigs	488	
Contig N50 length (Mb)	0.9	
Number of scaffolds	112	
Scaffold N50 length (Mb)	9.5	
Longest scaffold (Mb)	23.4	
Genome annotation		
Number of protein-coding genes	10,856	
Number of non-coding genes	3,362	
Number of gene transcripts	25,172	

* Assembly metric benchmarks are adapted from column VGP-2020 of “Table 1: Proposed standards and metrics for defining genome assembly quality” from ( Rhie *et al.*, 2021). ** BUSCO scores based on the hymenoptera_odb10 BUSCO set using v5.3.2. C = complete [S = single copy, D = duplicated], F = fragmented, M = missing, n = number of orthologues in comparison. A full set of BUSCO scores is available at https://blobtoolkit.genomehubs.org/view/Dolichovespula%20saxonica/dataset/CAJVQX01.1/busco.

**Table 2.** Chromosomal pseudomolecules in the genome assembly of *Dolichovespula saxonica*, iyDolSaxo1.

INSDC accession	Chromosome	Length (Mb)	GC%
OU426993.1	1	23.38	35.7
OU426994.1	2	13.34	35.2
OU426995.1	3	11.46	35.5
OU426996.1	4	10.81	35.8
OU426997.1	5	10.43	36.2
OU426998.1	6	10.04	35.7
OU426999.1	7	9.63	36.4
OU427000.1	8	9.59	35.8
OU427001.1	9	9.53	35.8
OU427002.1	10	9.51	36.2
OU427003.1	11	9.4	35.1
OU427004.1	12	8.12	35.1
OU427005.1	13	6.24	35.6
OU427006.1	14	5.94	35.4
OU427007.1	15	5.83	34.6
OU427008.1	16	5.81	37
OU427009.1	17	5.8	32.3
OU427010.1	18	5.48	35.3
OU427011.1	19	5.00	34.8
OU427012.1	20	4.66	35.2
OU427013.1	21	4.62	35
OU427014.1	22	4.61	36.3
OU427015.1	23	4.53	36
OU427016.1	24	4.09	36.9
OU427017.1	25	3.79	37.6
OU427018.1	26	3.65	35
OU427019.1	MT	0.02	16.3

The estimated Quality Value (QV) of the final assembly is 49.2 with *k*-mer completeness of 99.96%, and the assembly has a BUSCO v5.3.2 completeness of 96.3% (single = 95.2%, duplicated = 1.1%), using the hymenoptera_odb10 reference set ( *n* = 5,991).

Metadata for specimens, spectral estimates, sequencing runs, contaminants and pre-curation assembly statistics can be found at https://links.tol.sanger.ac.uk/species/85443.

## Genome annotation report

The *Dolichovespula saxonica* genome assembly (GCA_911387935.1) was annotated using the Ensembl rapid annotation pipeline ( Table 1; https://rapid.ensembl.org/Dolichovespula_saxonica_GCA_911387935.1/Info/Index). The resulting annotation includes 25,172 transcribed mRNAs from 10,856 protein-coding and 3,362 non-coding genes.

## Methods

### Sample acquisition and nucleic acid extraction

A male *Dolichovespula saxonica* (specimen ID Ox000189, ToLID iyDolSaxo1) was netted in Wytham Woods, Oxfordshire, UK (latitude 51.77, longitude –1.33) on 2019-08-20. The specimen was collected and identified by Liam Crowley (University of Oxford) and preserved on dry ice.

DNA was extracted at the Tree of Life laboratory, Wellcome Sanger Institute (WSI). The iyDolSaxo1 sample was weighed and dissected on dry ice with tissue set aside for Hi-C sequencing. Head and thorax tissue was cryogenically disrupted to a fine powder using a Covaris cryoPREP Automated Dry Pulveriser, receiving multiple impacts. High molecular weight (HMW) DNA was extracted using the Qiagen MagAttract HMW DNA extraction kit. Low molecular weight DNA was removed from a 20 ng aliquot of extracted DNA using the 0.8X AMpure XP purification kit prior to 10X Chromium sequencing; a minimum of 50 ng DNA was submitted for 10X sequencing. HMW DNA was sheared into an average fragment size of 12–20 kb in a Megaruptor 3 system with speed setting 30. Sheared DNA was purified by solid-phase reversible immobilisation using AMPure PB beads with a 1.8X ratio of beads to sample to remove the shorter fragments and concentrate the DNA sample. The concentration of the sheared and purified DNA was assessed using a Nanodrop spectrophotometer and Qubit Fluorometer and Qubit dsDNA High Sensitivity Assay kit. Fragment size distribution was evaluated by running the sample on the FemtoPulse system.

RNA was extracted from remaining head and thorax tissue of iyDolSaxo1 in the Tree of Life Laboratory at the WSI using TRIzol, according to the manufacturer’s instructions. RNA was then eluted in 50 μl RNAse-free water and its concentration assessed using a Nanodrop spectrophotometer and Qubit Fluorometer using the Qubit RNA Broad-Range (BR) Assay kit. Analysis of the integrity of the RNA was done using Agilent RNA 6000 Pico Kit and Eukaryotic Total RNA assay.

### Sequencing

Pacific Biosciences HiFi circular consensus and 10X Genomics read cloud DNA sequencing libraries were constructed according to the manufacturers’ instructions. Poly(A) RNA-Seq libraries were constructed using the NEB Ultra II RNA Library Prep kit. DNA and RNA sequencing was performed by the Scientific Operations core at the WSI on Pacific Biosciences SEQUEL II (HiFi), Illumina HiSeq 4000 (RNA-Seq) and HiSeq X Ten (10X) instruments. Hi-C data were also generated from abdomen tissue of iyDolSaxo1 using the Arima2 kit and sequenced on the Illumina NovaSeq 6000 instrument.

### Genome assembly, curation and evaluation

Assembly was carried out with HiCanu ( Nurk *et al.*, 2020). One round of polishing was performed by aligning 10X Genomics read data to the assembly with Long Ranger ALIGN, calling variants with FreeBayes ( Garrison & Marth, 2012). The assembly was then scaffolded with Hi-C data ( Rao *et al.*, 2014) using SALSA2 ( Ghurye *et al.*, 2019). The assembly was checked for contamination and corrected using the gEVAL system ( Chow *et al.*, 2016) as described previously ( Howe *et al.*, 2021). Manual curation was performed using gEVAL, HiGlass ( Kerpedjiev *et al.*, 2018) and Pretext ( Harry, 2022). The mitochondrial genome was assembled using MitoHiFi ( Uliano-Silva *et al.*, 2023), which runs MitoFinder ( Allio *et al.*, 2020) or MITOS ( Bernt *et al.*, 2013) and uses these annotations to select the final mitochondrial contig and to ensure the general quality of the sequence.

A Hi-C map for the final assembly was produced using bwa-mem2 ( Vasimuddin *et al.*, 2019) in the Cooler file format ( Abdennur & Mirny, 2020). To assess the assembly metrics, the *k*-mer completeness and QV consensus quality values were calculated in Merqury ( Rhie *et al.*, 2020). This work was done using Nextflow ( Di Tommaso *et al.*, 2017) DSL2 pipelines “sanger-tol/readmapping” ( Surana *et al.*, 2023a) and “sanger-tol/genomenote” ( Surana *et al.*, 2023b). The genome was analysed within the BlobToolKit environment ( Challis *et al.*, 2020) and BUSCO scores ( Manni *et al.*, 2021; Simão *et al.*, 2015) were calculated.

Table 3 contains a list of relevant software tool versions and sources.

**Table 3.** Software tools: versions and sources.

Software tool	Version	Source
BlobToolKit	4.0.7	https://github.com/blobtoolkit/blobtoolkit
BUSCO	5.3.2	https://gitlab.com/ezlab/busco
FreeBayes	1.3.1-17-gaa2ace8	https://github.com/freebayes/freebayes
gEVAL	N/A	https://geval.org.uk/
HiCanu	2.1.1	https://github.com/marbl/canu
HiGlass	1.11.6	https://github.com/higlass/higlass
Long Ranger ALIGN	2.2.2	https://support.10xgenomics.com/genome-exome/software/ pipelines/latest/advanced/other-pipelines
Merqury	MerquryFK	https://github.com/thegenemyers/MERQURY.FK
MitoHiFi	2	https://github.com/marcelauliano/MitoHiFi
PretextView	0.2	https://github.com/wtsi-hpag/PretextView
purge_dups	1.2.3	https://github.com/dfguan/purge_dups
SALSA	2.2	https://github.com/salsa-rs/salsa
sanger-tol/genomenote	v1.0	https://github.com/sanger-tol/genomenote
sanger-tol/readmapping	1.1.0	https://github.com/sanger-tol/readmapping/tree/1.1.0

### Genome annotation

The Ensembl gene annotation system ( Aken *et al.*, 2016) was used to generate annotation for the *Dolichovespula saxonica* assembly (GCA_911387935.1). Annotation was created primarily through alignment of transcriptomic data to the genome, with gap filling via protein-to-genome alignments of a select set of proteins from UniProt ( UniProt Consortium, 2019).

### Wellcome Sanger Institute – Legal and Governance

The materials that have contributed to this genome note have been supplied by a Darwin Tree of Life Partner. The submission of materials by a Darwin Tree of Life Partner is subject to the **‘Darwin Tree of Life Project Sampling Code of Practice’**, which can be found in full on the Darwin Tree of Life website here. By agreeing with and signing up to the Sampling Code of Practice, the Darwin Tree of Life Partner agrees they will meet the legal and ethical requirements and standards set out within this document in respect of all samples acquired for, and supplied to, the Darwin Tree of Life Project. 

Further, the Wellcome Sanger Institute employs a process whereby due diligence is carried out proportionate to the nature of the materials themselves, and the circumstances under which they have been/are to be collected and provided for use. The purpose of this is to address and mitigate any potential legal and/or ethical implications of receipt and use of the materials as part of the research project, and to ensure that in doing so we align with best practice wherever possible. The overarching areas of consideration are:

• Ethical review of provenance and sourcing of the material

• Legality of collection, transfer and use (national and international) 

Each transfer of samples is further undertaken according to a Research Collaboration Agreement or Material Transfer Agreement entered into by the Darwin Tree of Life Partner, Genome Research Limited (operating as the Wellcome Sanger Institute), and in some circumstances other Darwin Tree of Life collaborators.

## Data Availability

European Nucleotide Archive: *Dolichovespula saxonica* (Saxon wasp). Accession number PRJEB45173; https://identifiers.org/ena.embl/PRJEB45173. ( Wellcome Sanger Institute, 2021) The genome sequence is released openly for reuse. The *Dolichovespula saxonica* genome sequencing initiative is part of the Darwin Tree of Life (DToL) project. All raw sequence data and the assembly have been deposited in INSDC databases. Raw data and assembly accession identifiers are reported in Table 1.

## Author information

Members of the University of Oxford and Wytham Woods Genome Acquisition Lab are listed here: https://doi.org/10.5281/zenodo.4789928.

Members of the Darwin Tree of Life Barcoding collective are listed here: https://doi.org/10.5281/zenodo.4893703.

Members of the Wellcome Sanger Institute Tree of Life programme are listed here: https://doi.org/10.5281/zenodo.4783585.

Members of Wellcome Sanger Institute Scientific Operations: DNA Pipelines collective are listed here: https://doi.org/10.5281/zenodo.4790455.

Members of the Tree of Life Core Informatics collective are listed here: https://doi.org/10.5281/zenodo.5013541.

Members of the Darwin Tree of Life Consortium are listed here: https://doi.org/10.5281/zenodo.4783558.
