# Supplementary material for: The genome sequence of the Saxon Wasp, Dolichovespula saxonica (Fabricius, 1793)
Source: Wellcome Open Res. 2023 Oct 27;8:498. [Version 1] doi: 10.12688/wellcomeopenres.20180.1 (PMC10873546; doi:10.12688/wellcomeopenres.20180.1)
Supplement: Peer review history

# Reviewer report: Reviewer response for version 1

**DOI:** 10.21956/wellcomeopenres.22343.r72985

**Published:** 2024-02-27

**Authors:** Rodolpho Menezes

**Affiliations:** 1 Department of Biological Sciences, State University of Santa Cruz, Ilhéus, Brazil

The manuscript by Liam Crowley presents a genome assembly from a male of the saxon wasp, *Dolichovespula saxonica*. The final assembly is 221.8 Mb in 112 sequence scaffolds. Overall the manuscript presents an advance in the genomic field of social wasps. This genome information will be interesting for comparative genomics investigation within Vespidae and also for evolution and conservation studies. The methodology is thorough and explained and the figures are well constructed and the manuscript is well written. However, I think the author should compare its results with the assembled genomes from other wasp species. Moreover, since the author assembled the mitochondrial genome, I think it is interesting to show general (GC/AT content…) mitochondrial information about this species."

Are sufficient details of methods and materials provided to allow replication by others?

Yes

Is the rationale for creating the dataset(s) clearly described?

Yes

Are the datasets clearly presented in a useable and accessible format?

Yes

Are the protocols appropriate and is the work technically sound?

Yes

Reviewer Expertise:

Phylogenomics, Cytogenetics, and Phylogeography.

I confirm that I have read this submission and believe that I have an appropriate level of expertise to confirm that it is of an acceptable scientific standard.

# Reviewer report: Reviewer response for version 1

**DOI:** 10.21956/wellcomeopenres.22343.r72987

**Published:** 2024-02-16

**Authors:** Lucio Navarro-Escalante

**Affiliations:** 1 Department of Molecular Biosciences, The University of Texas at Austin, Austin, Texas, USA

**Overall**:

 The manuscript describes the sequencing, assembly and annotation of the genome of the wasp *Dolichovespula* *saxonica*. This work shows a high quality chromosome-level assembly obtained by combining PacBio HiFi reads, 10X Illumina PE reads and Hi-C scaffolding from a single male individual. The full sequence of its mitochondrial genome is also reported. The manuscript is well written, clear and includes all the necessary methodological details. All raw data is publicly available as well. Tables and figures are well explanatory and relevant. This genome assembly report seems to be an important contribution for improving biological understanding of members within the Vespidae wasp family.  Despite one minor comment, I support the indexing of this report and its data.

**Minor comment**:

 In the “Genome sequence report” section, authors should include that the number of chromosome-level scaffolds obtained agrees with previous karyotype analysis by Hoshiba et al. (1989).

**Reference**:

 Hoshiba H, Matsuura M, Imai HT: Karyotype Evolution in the Social Wasps Hymenoptera, Vespidae. JPN J Genet. 1989;64(3):209–22. 10.1266/jjg.64.209 ^1^

Are sufficient details of methods and materials provided to allow replication by others?

Yes

Is the rationale for creating the dataset(s) clearly described?

Yes

Are the datasets clearly presented in a useable and accessible format?

Yes

Are the protocols appropriate and is the work technically sound?

Yes

Reviewer Expertise:

Insect Genomics, Insect-Bacteria Symbiosis

I confirm that I have read this submission and believe that I have an appropriate level of expertise to confirm that it is of an acceptable scientific standard.

## References

[1] : Karyotype evolution in the social wasps (Hymenoptera, Vespidae). *The Japanese Journal of Genetics* .1989;64(3) : 10.1266/jjg.64.209 209-222 10.1266/jjg.64.209

# Reviewer report: Reviewer response for version 1

**DOI:** 10.21956/wellcomeopenres.22343.r72979

**Published:** 2024-02-16

**Authors:** Natalia Araujo

**Affiliations:** 1 Unit of Evolutionary Biology & Ecology –, Universite Libre de Bruxelles, Brussels, Brussels, Belgium

This is a data note reporting the assembly of the nuclear and mitochondrial genomes of the wasp *Dolichovespula saxonica *available at NCBI since July 2021. One single male was used for multiple sequencing strategies (genome sequencing, Hi-C, and transcriptomic data). The methods used for the assembly were the default pipeline of the DToL project, which is well accepted and known to result in good quality and complete genome assemblies. As the current reported assembly.

 I have no major comments about this report, only a few minor suggestions that do not reduce the overall quality of the manuscript:

 - On Table 1 the assembly release date has one extra 0 that should be deleted (from 20201-07-21 to 2021-07-21)

 - In the "Genome annotation report" section there is only information about the annotation of the nuclear genome. What were the results of the mtcontig annotation?

 - In the Methods, section "Genome assembly, curation and evaluation" it says "The mitochondrial genome was assembled using MitoHiFi ( Uliano-Silva *et al.*, 2023), which runs MitoFinder ( Allio *et al.*, 2020) or MITOS ( Bernt *et al.*, 2013)...", which one (MitoFinder or MITOS) was used to annotate the mtcontig of *D.* *saxonica*?

Are sufficient details of methods and materials provided to allow replication by others?

Yes

Is the rationale for creating the dataset(s) clearly described?

Yes

Are the datasets clearly presented in a useable and accessible format?

Yes

Are the protocols appropriate and is the work technically sound?

Yes

Reviewer Expertise:

Insects genomics and evolution

I confirm that I have read this submission and believe that I have an appropriate level of expertise to confirm that it is of an acceptable scientific standard.
